# Emergence and Containment of Canine Influenza Virus A(H3N2), Ontario, Canada, 2017–2018

**DOI:** 10.3201/eid2510.190196

**Published:** 2019-10

**Authors:** J. Scott Weese, Maureen E.C. Anderson, Yohannes Berhane, Kathleen F. Doyle, Christian Leutenegger, Roxanne Chan, Michelle Chiunti, Katerina Marchildon, Nicole Dumouchelle, Theresa DeGelder, Kiera Murison, Catherine Filejksi, Davor Ojkic

**Affiliations:** University of Guelph, Guelph, Ontario, Canada (J.S. Weese, K. Murison, D. Ojkic);; Ontario Ministry of Agriculture, Food and Rural Affairs, Guelph (M.E.C. Anderson);; National Centre for Foreign Animal Disease, Canadian Food Inspection Agency, Winnipeg, Manitoba, Canada (Y. Berhane);; Chidiac Animal Hospital Gravenhurst, Gravenhurst, Ontario, Canada (K.F. Doyle);; IDEXX Laboratories, West Sacramento, California, USA (C. Leutenegger); IDEXX Laboratories, Markham, Ontario, Canada (R. Chan);; Northumberland Veterinary Services, Colborne, Ontario, Canada (M. Chiunti);; Lake Country Animal Hospital, Severn, Ontario, Canada (K. Marchildon);; Fort Malden Animal Hospital, Amherstburg, Ontario, Canada (N. Dumouchelle);; Forest Glade Animal Hospital, Windsor, Ontario, Canada (T. DeGelder);; Ontario Ministry of Health and Longterm Care, Toronto, Ontario, Canada (C. Filejski)

**Keywords:** influenza, canine influenza virus A(H3N2), infectious disease outbreaks, canine diseases, infection control, Ontario, Canada, viruses

## Abstract

Canine influenza virus (CIV) A(H3N2) was identified in 104 dogs in Ontario, Canada, during December 28, 2017–October 30, 2018, in distinct epidemiologic clusters. High morbidity rates occurred within groups of dogs, and kennels and a veterinary clinic were identified as foci of infection. Death attributable to CIV infection occurred in 2 (2%) of 104 diagnosed cases. A combination of testing of suspected cases, contact tracing and testing, and 28-day isolation of infected dogs was used, and CIV transmission was contained in each outbreak. Dogs recently imported from Asia were implicated as the source of infection. CIV H3N2 spread rapidly within groups in this immunologically naive population; however, containment measures were apparently effective, demonstrating the potential value of prompt diagnosis and implementation of CIV control measures.

Canine influenza virus (CIV) is a regional cause of disease in dogs that has emerged from other host species of influenza A viruses (IAVs) as a result of adaptation and subsequent transmission within the naive dog population (*1*,*2*). Two main CIV strains are currently recognized. CIV A(H3N8) is an equine-origin virus that was identified in dogs in the United States in the early 2000s (*1*) but that is rarely identified now. In contrast, CIV H3N2 emerged in Asia from an avian influenza virus (H3N2) (*2*,*3*) and can be found in different regions in Asia (*2*–*4*). It was subsequently introduced to the United States on multiple occasions through importation of dogs from South Korea and China (*5*,*6*). Within an immunologically naive canine population, CIV can spread widely when introduced to a new dog population, and result in widespread illness and sporadic death. There is also some concern about the potential for CIV H3N2 to recombine with other IAVs (*7*), including human IAVs, potentially resulting in antigenic shift and creating relatively novel IAVs with broader host range and pandemic potential.

Novel CIVs are of concern for canine health because of the naive population and potential for rapid and widespread transmission. International, including transcontinental, movement of dogs is common, and CIV is one of many pathogens that can accompany transported dogs. In 2015, CIV H3N2 was introduced into the United States through the importation of dogs from Asia; the virus continues to circulate in the canine population within the country from that or subsequent importations (*5*,*6*,*8*). Despite its presence in the United States, CIV H3N2 had not been identified in Canada until the end of 2017. We describe the introduction and containment of CIV H3N2 in Ontario, Canada.

## Outbreak Investigation

Clinical testing identified CIV H3N2 in Ontario in December 2017, prompting prospective surveillance and interventions. Initial clinical diagnoses were based on positive H3N2 PCR results from a commercial respiratory PCR panel (IDEXX Laboratories, https://ca.idexx.com). Subsequent testing was performed at the Animal Health Laboratory, University of Guelph (Guelph, ON, Canada), using IAV matrix gene real-time PCR (rPCR) testing of nasal or pharyngeal swab specimens or a repeat of the respiratory PCR panel test. H3N2-specific hemagluttination inhibition (HI) testing was performed at the Animal Health Diagnostic Center, Cornell University (Ithaca, NY, USA), on serum samples collected from dogs that were suspected to have been infected recently but whose PCR results might have been negative by the time testing was performed. After identification of cases (dogs with CIV-positive PCR results), contact tracing was performed, with potentially exposed dogs tested whenever possible. Longitudinal testing was performed whenever possible, and an attempt was made to get weekly samples from all positive dogs until 2 negative results were obtained. Partial sequencing of the hemagglutinin gene was performed as previously described (*9*).

### Outbreak Cluster 1

**Figure 1:**
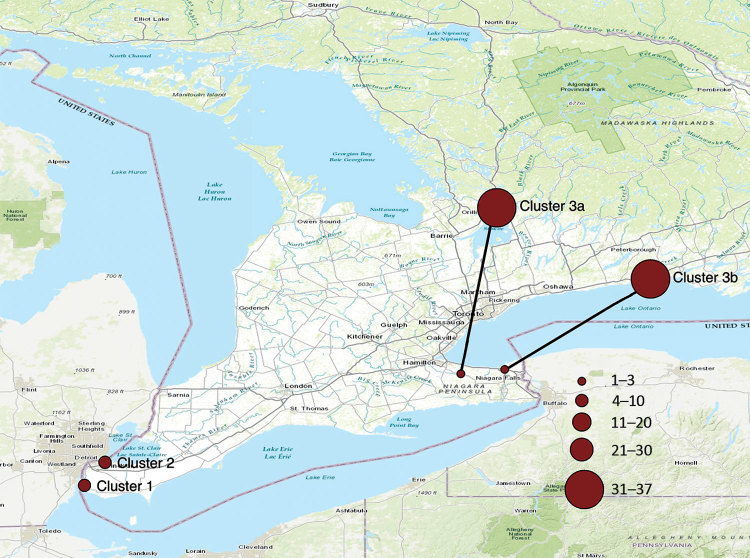
Approximate locations and number of dogs with diagnoses of canine influenza virus infection, Ontario, Canada, 2017–2018.

Two dogs received diagnoses of CIV H3N2 shortly after their arrival in southwestern Ontario (Figure 1) from South Korea. The dogs had been part of a larger group flown to Chicago, Illinois, USA; the 2 affected dogs were driven to Canada immediately after arrival. Both dogs had signs consistent with upper respiratory disease, characterized by fever, productive cough, and purulent nasal discharge. Samples were collected on December 28, 2017, within 24 hours of arrival in Canada.

The 2 imported dogs went to separate foster homes; all 6 canine contacts in those homes were identified on January 5, 2018, as infected with CIV H3N2. Two feline household contacts were negative. A 28-day isolation period was recommended; compliance was good, and no further cases were identified. Two dogs were clinically normal but still shedding CIV on January 18, but all were negative by January 31.

### Outbreak Cluster 2

This cluster occurred in a nearby community in southwestern Ontario; CIV H3N2 was identified in a dog with upper respiratory illness. Disease was first noted on January 20, 2018; samples were collected for an upper respiratory disease PCR panel on January 22. There was no known contact with dogs from cluster 1. The owner was a veterinary clinic employee who had handled a dog with severe and presumably infectious respiratory tract disease a few days before the onset of disease. That dog had died without testing being performed. Both canine household contacts developed respiratory disease that was diagnosed as CIV H3N2 from samples collected January 31.

The index case dog also had contact with a group of other dogs as part of a recurring canine group activity. Ten contacts were tested, and 1 dog tested positive. That dog developed mild respiratory disease; CIV H3N2 was detected on January 31.

Because the source of exposure of the index case was unclear, serologic testing was performed on the group of canine contacts in an attempt to determine whether other dogs might have been previously infected but had ceased shedding by the time of investigation. The only seropositive dogs were the affected household dogs and a single affected contact, which developed disease well after the index case.

The local public health unit implemented a mandatory 28-day confinement order for the affected dogs. No further cases were identified, and all dogs recovered uneventfully.

### Outbreak Cluster 3a

**Figure 2:**
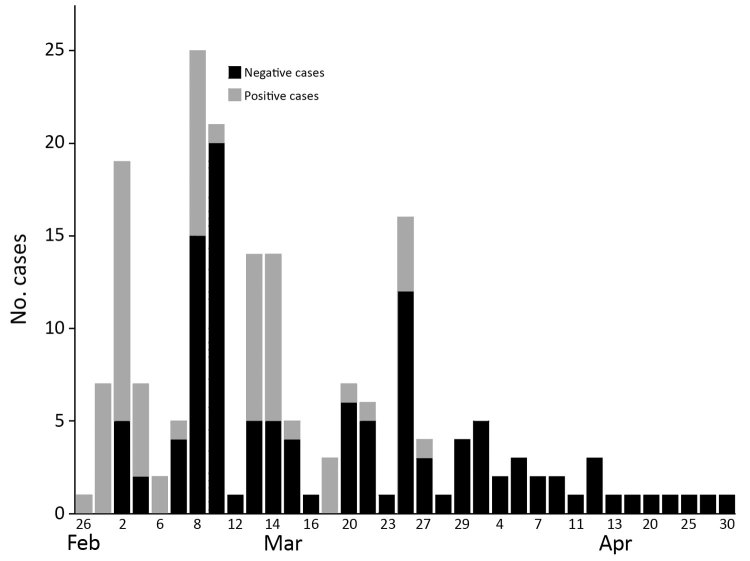
Time series of canine influenza diagnoses in clusters 3a and 3b, Ontario, Canada, 2017–2018. Transmission events within households are not depicted.

**Figure 3:**
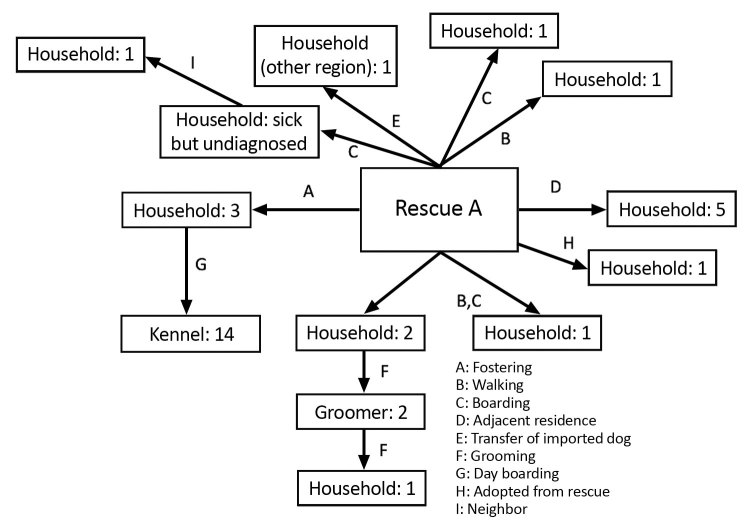
Suspected transmission pathways for canine influenza cluster 3a, Ontario, Canada, 2017–2018. Numbers in each box denote the number of confirmed (PCR positive) CIV infections.

This cluster was initially identified in central Ontario, starting February 26, 2018 (Figures 2, 3), during testing of a high-morbidity respiratory disease outbreak in a boarding kennel. Fourteen dogs at that kennel were infected.

Contact tracing raised suspicion of a rescue facility (rescue A) that had recently imported dogs from China as the source, because 1 of the imported dogs was fostered by the owner of 3 dogs that went to the affected kennel. Those 3 dogs developed upper respiratory tract disease on February 17, four days after contact with the imported dog. 

The rescue facility also offered boarding and dog walking services. One dog that was walked by rescue A tested positive for CIV (February 23 and March 1); CIV was then transmitted to a household canine contact, which transmitted CIV to a grooming service it visited. Two of the groomer’s own dogs were infected; 1 was euthanized because of severe respiratory disease. One additional dog was then infected at the groomer’s.

Other cases linked to the rescue facility were identified, including 5 dogs (diagnosed March 2 and 5) whose owner resided adjacent to rescue A, 1 dog (diagnosed March 2) that had boarded at the rescue during February 17–24, a dog that had been at rescue A for day care, another dog that had visited for dog walking (diagnosed March 3), and a dog that became sick shortly after being adopted from the rescue facility. One additional case without a known origin was identified in a dog that developed respiratory disease around March 12; CIV was diagnosed on March 19. This dog had no known contact with other affected dogs but was regularly walked along a public trail. Follow-up information was not available for the remaining 3 infected dogs.

Another linked case was identified in a different city ≈300 km away. A dog imported from China in the same shipment that went to rescue A was transferred to another group (rescue B); respiratory disease subsequently developed in an unclear number of contacts. Nasal swab specimens were collected from 15 dogs associated with rescue B on March 9, and 1 was CIV positive. It was suspected that other dogs were also infected but had ceased shedding by the time of sampling (24 days after the imported dog arrived), given the delay in identifying this group.

With 9 separate epidemiologic links and a history of importation of dogs from China, rescue A was the presumed source of CIV H3N2. It had received imported dogs from China on February 13. Upper respiratory tract disease was reported in 42/64 (66%) dogs at the rescue during February 16–March 2. Testing of 10 dogs at the facility was performed until March 8, at which point none of the dogs were found to be shedding CIV. One death attributable to respiratory disease was reported at rescue A, but testing was not performed.

A 28-day isolation period was recommended for all infected dogs; owners were receptive to isolation and infection control recommendations. The affected boarding kennel was closed until all dogs tested negative. The affected grooming facility was also closed until the owner’s dogs tested negative and the facility had been cleaned and disinfected.

### Outbreak Cluster 3b

**Figure 4:**
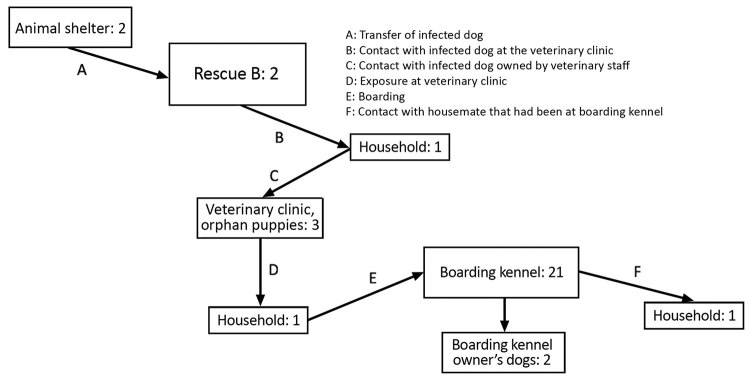
Suspected transmission pathways for canine influenza cluster 3b, Ontario, Canada, 2017–2018. Numbers in each box denote the number of confirmed (PCR positive) CIV infections.

A secondary cluster was identified in eastern Ontario, ≈250 km from the central Ontario outbreak. The first diagnosed case was from a group of 3 orphaned puppies that were being cared for by personnel from a veterinary clinic, but infection was ultimately linked to a dog (dog A) from a humane society ≈250 km away (Figure 4). A local rescue group (rescue C) had obtained dog A from a shelter in the Niagara, Ontario, region (≈300 km away from cluster 3a and 250 km from the veterinary clinic, but only 25 km from where 1 of the imported dogs was transferred). Dog A was surrendered to the shelter on February 12 and transferred to rescue C on February 21. This dog was taken to the veterinary clinic after arrival at rescue C, at which time nasal discharge, sneezing, and coughing were noted. Testing was not performed, and the dog was not available for testing until March 18. Serologic testing was performed because of the time that had passed, and the dog was seropositive (hemagglutinin inhibition [HI] titer 1:256; >1:16 indicates exposure). Because this dog had no history of travel outside Canada or CIV vaccination, this result provided a presumptive CIV diagnosis. Two dogs at rescue C that developed respiratory disease after dog A arrived were also seropositive on samples collected March 18; HI titers were 1:64 and 1:1,024.

Furthermore, it was reported that an outbreak of upper respiratory tract disease had occurred in dogs at rescue C shortly after dog A left. Five dogs at the shelter were tested by PCR; however, testing occurred March 9, which was 16 days after departure of the affected dog, and all tests were negative. Serologic testing performed on 1 of those dogs was positive (HI titer of 1:1,024), supporting the presence of a CIV H3N2 cluster in that shelter, with transmission to rescue C by dog A.

The origin of CIV was unclear. Given the timing of arrival at the shelter and onset of disease, it is possible that dog A was infected at the shelter or shortly before arrival. No source of infection was identified; however, the dog was surrendered to the shelter from an area close to rescue B at the same time as the localized cluster associated with that group was active.

Dog A was at the veterinary clinic at the same time as another dog (dog B) owned by an employee of the clinic. CIV was diagnosed in dog B on February 28, and dog B’s 5 household canine contacts were subsequently infected. Prior to the onset of signs of respiratory disease, dog B attended the veterinary clinic with its owner when the litter of 3 orphan puppies was in the clinic. The puppies started showing signs of respiratory tract disease on March 2; a sample was collected from 1 puppy the next day, and CIV was diagnosed by PCR.

One dog that had been at the clinic for elective surgery was infected. That dog then visited a local doggy day care. Cough was noted on the first day of stay (March 6), and the dog visited the facility for the next week. Twenty-one dogs that visited the facility were infected, along with 2 of the facility owner’s dogs. A 15-year-old dog with renal disease died, with the cause attributed to CIV. The kennel was closed to all but infected dogs for the next 3 weeks and underwent thorough cleaning and disinfection; 28-day isolation of all exposed or infected dogs was recommended. To reduce the number of potential exposed animals and facilitate cleaning and disinfection, the veterinary clinic temporarily restricted elective admissions for 1 week.

### Outbreak Cluster 4

In October 2018, another 2 infected dogs were identified. Both had had contact with rescue A, and it was subsequently learned that another group of dogs had been imported from China ≈3 weeks earlier. Seven H3N2 infections among nonimported dogs were then confirmed at rescue A. Five additional H3N2-infected dogs were identified, all of which had contact with rescue A through boarding or walking. Sixty-two dogs were tested as part of the investigation and response, and no further cases were identified. Nine of the infected dogs had been vaccinated against CIV H3N2 in the spring and had received the required 2-dose initial series.

### Other Cases

During the time of cluster 3, another potential case was identified in a town >200 km from the nearest affected area. The dog had mild respiratory tract disease; matrix gene rPCR testing yielded a result at the upper end of the inconclusive range (40 cycles; reference range, positive <36 cycles, inconclusive >36 but *<*40). An affected housemate was negative, and there were no reported contacts with imported dogs or dogs from affected areas. Therefore, a false-positive result was suspected.

### Sequence Analysis

Partial hemagglutinin (H) gene was amplified from 32 samples that tested positive on matrix gene rPCR for Sanger sequencing. One sample each were from clusters 1 and 2, and those samples were identical. All 20 samples from cluster 3a were genetically identical and were 99.7% identical to clusters 1 and 2. Five samples from cluster 3b were identical and were 99.5% identical to clusters 1 and 2. Three samples from cluster 3b were identical to those from cluster 3a; all 3 were from dogs in the first group of dogs in cluster 3b, suggesting that genetic drift occurred between the dogs in this household and the later cases.

### Subsequent Surveillance

Overall, 104 infected dogs were identified during December 28, 2017–October 30, 2018; however, it is likely that many more dogs were infected, as testing of all affected dogs in a group was not always performed. As part of this investigation, tested 263 dogs.

Cluster 3 was considered to be over on May 1, which was 28 days after the last known positive result. No additional cases of CIV were identified in Ontario until October 16, 2018, when cluster 4 was identified. Because a regulatory requirement for veterinarians and diagnostic laboratories to report novel influenza (which would include CIV) infection in animals to local public health units went into effect on January 1, 2018, it is likely that all diagnosed cases were identified. Although only a subset of dogs with respiratory disease are currently tested for CIV, the high infection rate of CIV would be expected to result in a greater impetus to test, given the obvious clusters of disease that can occur. Although we cannot state definitely that CIV was eradicated, no infections were identified for at least 6 months after the final cases ceased shedding CIV.

### Outcome and Duration of Shedding

Deaths attributed to CIV were reported in 2 (2%) of the diagnosed cases, both of which were in older dogs with underlying diseases. CIV was also suspected in at least 1 additional death.

Multiple samples were collected from 44 CIV-positive dogs (range 2–5 samples, median 2). Of the 22 (50%) that had >1 positive sample, the interval between the 2 positive results, representing the minimal shedding period, ranged from 4 to 20 days (median 12 days). The duration from first positive to first negative ranged from 4 to 30 days (median 18 days). Two dogs had a negative sample between 2 positive samples.

### Human Surveillance

In all clusters, public health units requested that potentially exposed persons report influenza-like illness so that testing could be performed. No human illnesses were reported from the undefined number of exposed human contacts.

## Discussion

Multiple introductions of CIV H3N2 have occurred in Ontario, Canada, with subsequent transmission within the canine population. A unique aspect of this investigation was the degree of contact tracing and active surveillance, such that a source of exposure was identified for almost all CIV-positive dogs. The source of infection was discerned for all dogs identified after the index cases in clusters 1, 2, 3b, and 4. In cluster 3a, 1 case had no known exposure, but the dog had been walked on public trails. No information was available for 3 dogs; contact tracing identified clear or plausible links for all other cases.

The first cluster was clearly associated with importation from South Korea, because the index cases were in dogs that had been imported within days of diagnosis. The origin of the second cluster is unclear. The timing, location, and sequence data suggest a link to cluster 1, but a separate introduction cannot be excluded. The third and fourth clusters presumably originated from separate groups of dogs imported from China, given the number of epidemiologic links to that facility and the timing and sequence data.

Although virtually the entire dog population of Canada is presumably immunologically naive to CIV H3N2 because of a lack of previous exposure and very low CIV vaccination rates, this highly transmissible virus was contained. Illness rates were high within groups, but a relatively small number of distinct groups was infected. It is possible that other clusters of disease were ongoing, but the high illness rate associated with CIV, the high awareness through media reports and other communications when the outbreak was under way, and the reportable nature of the disease suggest that unidentified clusters (at least large clusters) are unlikely.

It was encouraging that CIV H3N2 was apparently eradicated multiple times. Although it is impossible to determine what actions were effective, the combination of rapid response, active surveillance of contacts, widespread communication, voluntary closure of affected facilities, and 28-day isolation of infected and exposed dogs appeared to contain this highly transmissible virus, even in an immunologically naive population. Time of year might have facilitated control; the cases occurred during periods of cold or otherwise inclement weather, something that likely reduced contact between dogs during walking, visits to parks, and similar activities. The ability to control these CIV introductions is in contrast to reports from the United States, where larger, more sustained outbreaks have occurred, perhaps largely the result of a lack of a coordinated effort to identify and contain the disease. Although the cost–benefit ratio of CIV containment can be debated, the experience in Ontario suggests that an active approach can be effective, provided appropriate personnel and resources are available.

Information about CIV H3N2 mortality rates is limited. The 2% mortality rate reported here is consistent with the 2.5% (1/40) rate reported in a metropolitan US outbreak (*8*). Mortality rates for CIV can be overestimated if animals with serious illness are more likely to be tested; however, testing bias is less of a concern in this study, given the scope of the investigation. Both dogs that died were older animals, consistent with the presumed increased risk in this population. Although the true mortality rate may be lower because of lack of testing of mildly affected animals, serious respiratory disease, including death, can occur.

A bivalent CIV H3N2/H3N8 vaccine was available in Canada during the period of this investigation, but vaccination coverage is anecdotally very low because of the foreign nature of CIV. Vaccination played little role in containment of these outbreaks. Few dogs from affected areas were vaccinated, and the need for 2 doses, 14–28 days apart, reduces the potential for vaccination to help during active outbreaks. Nevertheless, vaccination of dogs in affected and adjacent communities was recommended to reduce the risk of continued spread if initial containment had failed. Most infected dogs in cluster 4 had been properly vaccinated against CIV H3N2. This raises concern about vaccine efficacy; however, interpretation is difficult because CIV vaccines are labeled as an aid for control of disease, focused on reduction of severity of disease. It is possible that vaccination reduced severity of disease. These data do not mean that vaccination is not warranted but serve as a reminder that CIV vaccination cannot be relied on as a tool to prevent entrance or dissemination of CIV in dog populations.

Sun et al. expressed concern about the potential that dogs could act as mixing vessels between human and nonhuman influenza viruses (*7*). No humans were tested as part of the surveillance of these canine cases. Response varied by health unit, but human contacts of infected dogs were generally asked to report whether they developed signs and symptoms consistent with influenza. Despite widespread human exposure in the ongoing CIV H3N2 outbreak in the United States, human cases have not been identified, suggesting that the public health risk from CIV H3N2 is limited. However, because dogs are susceptible to some influenza viruses, such as pandemic influenza A(H1N1) (*10*), co-infection of dogs and humans in households raises concern about the potential for reassortment.

In summary, CIV H3N2 is a highly transmissible virus with the potential to cause high-morbidity outbreaks, along with economic and social disruption in the canine and veterinary industries. Even though human health risks are believed to be low, endemic circulation of a nonhuman influenza A virus, particularly in an animal population with such close human contact, raises concern. Clarifying transmission routes and control options is critical for CIV control; this outbreak demonstrates the likely effectiveness of a concerted approach focusing on testing, contact tracing, voluntary isolation, and communication.
